# Association between 24h Urinary Sodium and Potassium Excretion and Estimated Glomerular Filtration Rate (eGFR) Decline or Death in Patients with Diabetes Mellitus and eGFR More than 30 ml/min/1.73m^2^

**DOI:** 10.1371/journal.pone.0152306

**Published:** 2016-05-02

**Authors:** Takanobu Nagata, Hiroshi Sobajima, Norimi Ohashi, Akihiro Hirakawa, Takayuki Katsuno, Yoshinari Yasuda, Seiichi Matsuo, Naotake Tsuboi, Shoichi Maruyama

**Affiliations:** 1 Department of Nephrology, Nagoya University Graduate School of Medicine, Nagoya, Japan; 2 Department of Diabetology and Nephrology, Ogaki Municipal Hospital, Ogaki, Japan; 3 Center for Advanced Medicine and Clinical Research, Nagoya University Hospital, Nagoya, Japan; The University of Tokyo, JAPAN

## Abstract

**Background:**

Data regarding the association between 24h urinary sodium and potassium excretion with kidney outcomes in patients with diabetes mellitus is currently scarce.

**Methods:**

We conducted a single-center, retrospective cohort study in which 1230 patients with diabetes who had undergone a 24h urinary sodium and potassium excretion test were analyzed. Patients with incomplete urine collection were excluded based on 24h urinary creatinine excretion. Outcomes were the composite of a 30% decline in eGFR or death. Multivariate cox regression analysis was used to investigate the association between urinary sodium and potassium excretion and outcomes.

**Results:**

With a mean follow up period of 5.47 years, 130 patients reached the outcomes (30% decline in eGFR: 124, death: 6). Mean (SD) eGFR and 24h urinary sodium and potassium excretion at baseline were 78.6 (19.5) ml/min/1.73m^2^, 4.50 (1.64) g/day, and 2.14 (0.77) g/day. Compared with sodium excretion < 3.0 g/day, no significant change in risk of outcomes was observed with increased increments of 1.0 g/day. Compared with potassium excretion of < 1.5 g/day, 2.0–2.5 g/day, and 2.5–3.0 g/day were significantly associated with a lower risk of outcomes (hazard ratio [HR], 0.49 and 0.44; 95% confidence interval [CI], 0.28 to 0.84 and 0.22 to 0.87).

**Conclusions:**

24h urinary sodium excretion was not significantly associated with a risk of 30% decline in eGFR or death in patients with diabetes. However, an increased risk of 30% decline in eGFR or death was significantly associated with 24h urinary potassium excretion < 1.5 g/day than with 2.0–2.5 g/day and 2.5–3.0 g/day.

## Introduction

The prevalence of chronic kidney disease (CKD) is a major public health issue worldwide[1]. Diabetes mellitus is the leading cause of CKD, and therefore, prevention of occurrence or progression in patients with diabetes is an important clinical concern.

Previous studies have shown that high sodium intake leads to hypertension or incidence of cardiovascular disease (CVD) in healthy individuals, patients with hypertension, and those with a history of CVD and diabetes[2–4]. Thus, the American Diabetes Association recommends restricting sodium intake to <2.3 g/day in patients with diabetes[5], while KDIGO guidelines recommend <2 g/day in patients with CKD[1]. However, several observational or prospective studies have reported that not all patients (such as those with high cardiovascular risk or diabetes) benefit from sodium restriction[2,6–9]. Furthermore, several studies have shown that the amount of urinary sodium excretion (as a substitute for intake) is not significantly associated with kidney outcomes[8,10–12].

Increased potassium intake has many beneficial effects on blood pressure (BP)[3,13] and risk of CVD[2,4]. The World Health Organization’s guidelines recommend a dietary potassium intake of >3.51 g/day in adults[14], while the Kidney Disease Outcomes Quality Initiative recommends >4 g/day in patients with CKD stages 1 to 2 and 2–4 g/day in patients with CKD stages 3 to 4[15]. The majority of reports have indicated that increased urinary potassium excretion is associated with a reduced risk for kidney outcomes[8,12,16]. However, recent reports have shown that high urinary potassium excretion is associated with an increased risk of halving the estimated glomerular filtration rate (eGFR) or end-stage renal disease (ESRD) in patients with CKD[17]. These inconsistent results could be attributed to differing outcome measures, patients’ backgrounds, methods for estimation of 24h urinary excretion (spot urine or 24h urine collection), or adjusted variables. At present, data regarding the association between 24h urinary sodium and potassium excretion and kidney outcomes in patients with diabetes is scarce.

The aim of this study was to assess the association between 24h urinary sodium and potassium excretion and kidney outcomes in a single-center, retrospective cohort of patients with diabetes.

## Materials and Methods

### Patients

This was a retrospective and observational cohort study at a single diabetes center in Ogaki Municipal Hospital, Ogaki, Japan. This is a tertiary hospital and a major diabetic referral center in the medical district with a population of approximately 400,000. All patients with diabetes admitted to the center are generally recommended to undergo a 24h urine collection test at least once for the purposes of accurate diagnosis of diabetic nephropathy and evaluation of insulin secretion capacity and dietary status. All patients received standard medical care including personalized glycemic, blood pressure, and lipid control in accordance with recommended guidelines.

Patients with diabetes who underwent the 24h urine collection test between January 1, 2007 and December 31, 2011, were enrolled in this study. The actual time of the test was considered as the baseline.

Exclusion criteria included: (i) eGFR < 30 ml/min/1.73m^2^; (ii) incomplete urine collection, defined as urine creatinine excretion deviation by ±25% of the predicted value as calculated by age, sex, height, and weight[18][19]; (iii) unavailable data for urinary albumin, sodium, and potassium excretion; (iv) no follow up of serum creatinine at Ogaki Municipal Hospital after the 24h urine collection test was performed; (v) in-hospital 24h urine collection test; (vi) comorbidity with autoimmune disease, glomerulonephritis, cancer, or liver cirrhosis at baseline, where the disease or treatment could affect kidney function; and (vii) hospital-based treatment duration of less than six months prior to baseline. This final exclusion criterion was included, as it generally requires six months to complete initial patient education, treatment modification, and evaluation of diabetic complications or other comorbidities.

This study protocol was approved by the ethics committee at Ogaki Municipal Hospital according to the Declaration of Helsinki. This study was conducted using a linkable, anonymous data set. The waiver of informed consent provided to patients was approved by the ethics committee.

### Outcomes

The primary outcome was a 30% decline in eGFR from baseline or death due to any causes. Where diseases listed under exclusion criteria occurred during follow up, patients were considered a censored case at the time of diagnosis.

### Data collection

Baseline characteristics were collected from patients’ medical record. Serum creatinine was measured using an enzymatic method. The eGFR was calculated using the equation generated by the Japanese Society of Nephrology: eGFR (ml/min/1.73 m^2^) = 194 × Serum creatinine^-1.094^ × Age^-0.287^ × 0.739 (for female patients)[20]. Blood and urine laboratory studies were performed as part of patients’ standard medical care. Follow-up data and outcomes were collected from patients’ medical records. Patients were studied until the following end points: a 30% decline in eGFR, death, lack of follow-up, or study end point (June 30, 2015)

### Statistical analysis

Continuous variables were expressed as the mean ± standard deviation [SD] and categorical variables were expressed as the number and proportion. Inter-group comparisons of baseline characteristics were performed using one-way analysis of variance for continuous variables and the chi-squared test for categorical variables. We evaluated the association between 24h urinary sodium (or potassium) excretion and the primary outcome based on the Cox model as a restricted cubic spline function with 4 knots (located at the 5th, 35th, 65th, and 95th percentiles)[21]. We further categorized 24h urinary sodium excretion into 5 groups (<3.0 g/day, 3.0–4.0 g/day, 4.0–5.0 g/day, 5.0–6.0 g/day, and >6.0 g/day), while 24h urinary potassium excretion was similarly classified (<1.5 g/day, 1.5–2.0 g/day, 2.0–2.5 g/day, 2.5–3.0 g/day, and >3.0 g/day). Less than 3.0 g/day for sodium excretion and <1.5 g/day for potassium excretion were selected as the reference categories and univariate and multivariate Cox regression analyses were performed. A P-value < 0.05 was considered statistically significant. All statistical analyses were performed using R (The R Foundation for Statistical Computing, Vienna, Austria, http://www.r-project.org, version 3.2.2).

## Results

**Fig 1:**
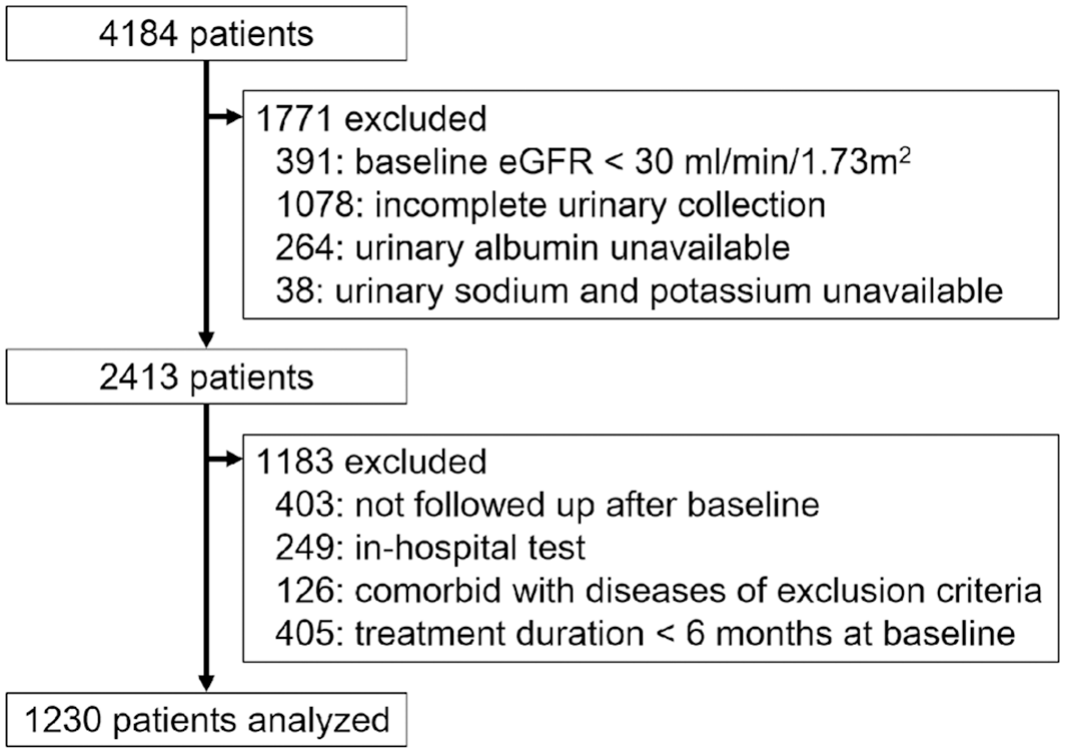
Flowchart of number of eligible patients after screening.

A total of 4184 patients were screened for this study, and 1230 eligible patients were finally included and analyzed (Fig 1). During screening, 1078 patients were excluded due to incomplete 24h urine collection from 3793 patients with an eGFR > 30 ml/min/1.73m^2^.

With a mean (SD) follow up period of 5.47 (2.28) years, 130 patients reached an outcome-based end point (30% decline of eGFR: n = 124, death: n = 6). Mean 24h sodium and potassium excretion at baseline were 4.50 (1.64) g/day and 2.14 (0.77) g/day, mean age was 62.2 (10.9) years, mean eGFR was 78.6 (19.5) ml/min/1.73m^2^, mean glycated hemoglobin was 7.26 (0.95) %, and mean 24h urinary albumin excretion was 44.4 (117.9) mg/day (Tables 1 and 2).

**Table 1:** Baseline characteristics categorized by 24h sodium excretion.

		24h urinary sodium excretion					
Variable	Overall	<3.0 g/day	3.0–4.0 g/day	4.0–5.0 g/day	5.0–6.0 g/day	>6.0 g/day	P-value
Number of patients	1230	220	274	333	222	181	
24h urinary sodium excretion, g/day	4.50 (1.64)	2.46 (0.48)	3.52 (0.27)	4.47 (0.30)	5.45 (0.29)	7.36 (1.35)	<0.001
Age, years	62.2 (10.9)	65.2 (9.9)	63.6 (10.3)	61.9 (10.6)	60.8 (11.7)	58.4 (11.1)	<0.001
Male, n (%)	651 (52.9)	92 (41.8)	133 (48.5)	169 (50.8)	127 (57.2)	130 (71.8)	<0.001
Height, cm	159.0 (9.1)	155.8 (8.5)	157.7 (9.2)	158.7 (8.6)	160.4 (8.6)	163.7 (9.3)	<0.001
Weight, kg	61.5 (11.6)	56.8 (9.8)	59.5 (11.1)	60.6 (10.0)	64.2 (11.3)	68.8 (13.6)	<0.001
BMI, kg/m^2^	24.2 (3.6)	23.3 (3.4)	23.8 (3.5)	24.0 (3.5)	24.9 (3.6)	25.5 (3.8)	<0.001
Systolic BP, mmHg	131.7 (17.5)	130.5 (18.6)	132.2 (18.0)	131.3 (17.4)	131.9 (16.9)	133.3 (16.3)	0.570
Diastolic BP, mmHg	72.6 (12.2)	71.0 (12.5)	71.5 (11.5)	72.2 (11.6)	73.0 (12.4)	76.7 (12.7)	<0.001
Type 2 diabetes, n (%)	1112 (90.4)	191 (86.8)	247 (90.1)	300 (90.1)	206 (92.8)	168 (92.8)	0.199
Diabetes duration, years	12.0 (8.0)	13.4 (8.6)	11.9 (8.0)	12.2 (8.3)	11.3 (7.3)	11.0 (7.5)	0.022
Diabetic retinopathy, n (%)	479 (38.9)	91 (41.4)	104 (38.0)	127 (38.1)	92 (41.4)	65 (35.9)	0.731
History of CVD, n (%)	250 (20.3)	64 (29.1)	57 (20.8)	59 (17.7)	45 (20.3)	25 (13.8)	0.002
Oral hypoglycemic agent, n (%)	818 (66.5)	132 (60.0)	180 (65.7)	225 (67.6)	150 (67.6)	131 (72.4)	0.119
Insulin, n (%)	408 (33.2)	88 (40.0)	87 (31.8)	110 (33.0)	73 (32.9)	50 (27.6)	0.116
RAAS blockade, n (%)	611 (49.7)	114 (51.8)	141 (51.5)	161 (48.3)	109 (49.1)	86 (47.5)	0.852
Calcium channel blocker, n (%)	456 (37.1)	93 (42.3)	108 (39.4)	112 (33.6)	81 (36.5)	62 (34.3)	0.237
Diuretics, n (%)	137 (11.1)	23 (10.5)	35 (12.8)	39 (11.7)	21 (9.5)	19 (10.5)	0.798
Statin, n (%)	440 (35.8)	81 (36.8)	106 (38.7)	119 (35.7)	73 (32.9)	61 (33.7)	0.688
Total protein, g/dl	7.48 (0.45)	7.51 (0.41)	7.50 (0.46)	7.45 (0.44)	7.50 (0.47)	7.46 (0.44)	0.434
Albumin, g/dl	4.39 (0.26)	4.37 (0.25)	4.40 (0.26)	4.38 (0.27)	4.40 (0.28)	4.43 (0.27)	0.138
Uric acid, mg/dl	5.07 (1.37)	5.21 (1.35)	5.08 (1.40)	4.93 (1.38)	5.07 (1.34)	5.14 (1.38)	0.171
Creatinine, mg/dl	0.72 (0.19)	0.74 (0.20)	0.72 (0.19)	0.72 (0.20)	0.71 (0.18)	0.72 (0.15)	0.422
eGFR, ml/min/1.73m^2^	78.6 (19.5)	73.1 (18.3)	76.9 (18.6)	78.6 (19.5)	82.1 (19.9)	83.6 (19.7)	<0.001
HbA1c, %	7.26 (0.95)	7.21 (0.92)	7.25 (0.99)	7.25 (0.93)	7.29 (0.96)	7.28 (0.97)	0.927
Serum sodium, mEq/l	139.6 (1.8)	139.7 (2.0)	139.7 (2.0)	139.6 (1.8)	139.50 (1.7)	139.4 (1.6)	0.376
Serum potassium, mEq/l	4.44 (0.37)	4.42 (0.38)	4.42 (0.36)	4.43 (0.39)	4.46 (0.38)	4.46 (0.34)	0.547
Serum chloride, mEq/l	104.3 (2.4)	104.6 (2.4)	104.4 (2.5)	104.4 (2.4)	104.1 (2.1)	103.9 (2.2)	0.028
Total cholesterol, mg/dl	198.8 (34.7)	199.4 (34.1)	199.5 (34.0)	199.0 (36.4)	197.8 (34.1)	198.2 (34.3)	0.980
HDL cholesterol, mg/dl	53.3 (14.0)	54.2 (15.0)	53.9 (12.9)	53.8 (14.3)	52.7 (14.6)	51.3 (12.6)	0.198
24h urinary albumin excretion, mg/day	44.4 (117.9)	38.5 (86.7)	40.1 (149.7)	48.5 (132.2)	51.1 (99.4)	42.6 (85.4)	0.719
24h urinary potassium excretion, g/day	2.14 (0.77)	1.71 (0.65)	1.97 (0.61)	2.11 (0.65)	2.35 (0.72)	2.74 (0.91)	<0.001

Categorical variables are expressed as the number (%) and continuous variables are expressed as the mean (SD).

BMI, body mass index; BP, blood pressure; CVD, cardiovascular disease; RAAS, renin-angiotensin-aldosterone system; eGFR, estimated glomerular filtration rate; HbA1c, glycated hemoglobin; HDL, high-density lipoprotein

**Table 2:** Baseline characteristics categorized by 24h potassium excretion.

		24h urinary potassium excretion					
Variable	Overall	<1.5 g/day	1.5–2.0 g/day	2.0–2.5 g/day	2.5–3.0 g/day	>3.0 g/day	P-value
Number of patients	1230	242	349	301	194	144	
24h urinary potassium excretion, g/day	2.14 (0.77)	1.23 (0.21)	1.75 (0.14)	2.24 (0.14)	2.72 (0.15)	3.64 (0.62)	<0.001
Age, years	62.2 (10.9)	63.4 (11.6)	61.3 (11.3)	62.6 (9.3)	61.1 (12.0)	62.7 (9.7)	0.100
Male, n (%)	651 (52.9)	109 (45.0)	164 (47.0)	181 (60.1)	111 (57.2)	86 (59.7)	<0.001
Height, cm	159.0 (9.1)	156.5 (9.5)	158.1 (8.9)	159.8 (8.8)	160.7 (8.7)	161.3 (9.4)	<0.001
Weight, kg	61.5 (11.6)	57.4 (9.4)	61.2 (11.9)	62.5 (10.7)	63.1 (12.0)	65.1 (14.1)	<0.001
BMI, kg/m^2^	24.2 (3.6)	23.4 (3.4)	24.4 (3.8)	24.4 (3.4)	24.3 (3.4)	24.7 (3.6)	0.003
Systolic BP, mmHg	131.7 (17.5)	131.6 (18.5)	133.2 (18.7)	129.9 (16.6)	131.3 (16.2)	133.0 (16.3)	0.156
Diastolic BP, mmHg	72.6 (12.2)	71.7 (12.9)	72.7 (12.7)	72.4 (11.7)	72.9 (10.7)	73.9 (12.6)	0.541
Type 2 diabetes, n (%)	1112 (90.4)	211 (87.2)	321 (92.0)	278 (92.4)	173 (89.2)	129 (89.6)	0.227
Diabetes duration, years	12.0 (8.0)	12.4 (8.6)	12.4 (8.2)	11.9 (7.8)	11.7 (7.5)	11.2 (7.7)	0.558
Diabetic retinopathy, n (%)	479 (38.9)	93 (38.4)	147 (42.1)	116 (38.5)	70 (36.1)	53 (36.8)	0.649
History of CVD, n (%)	250 (20.3)	50 (20.7)	81 (23.2)	69 (22.9)	34 (17.5)	16 (11.1)	0.021
Oral hypoglycemic agent, n (%)	818 (66.5)	154 (63.6)	242 (69.3)	201 (66.8)	130 (67.0)	91 (63.2)	0.576
Insulin, n (%)	408 (33.2)	89 (36.8)	118 (33.8)	92 (30.6)	59 (30.4)	50 (34.7)	0.520
RAAS blockade, n (%)	611 (49.7)	124 (51.2)	191 (54.7)	144 (47.8)	87 (44.8)	65 (45.1)	0.126
Calcium channel blocker, n (%)	456 (37.1)	99 (40.9)	135 (38.7)	109 (36.2)	67 (34.5)	46 (31.9)	0.384
Diuretics, n (%)	137 (11.1)	38 (15.7)	45 (12.9)	25 (8.3)	17 (8.8)	12 (8.3)	0.028
Statin, n (%)	440 (35.8)	88 (36.4)	136 (39.0)	96 (31.9)	79 (40.7)	41 (28.5)	0.062
Total protein, g/dl	7.48 (0.45)	7.47 (0.46)	7.44 (0.46)	7.50 (0.44)	7.48 (0.41)	7.54 (0.45)	0.254
Serum albumin, g/dl	4.39 (0.26)	4.35 (0.29)	4.37 (0.26)	4.41 (0.26)	4.44 (0.24)	4.43 (0.23)	<0.001
Serum uric acid, mg/dl	5.07 (1.37)	4.91 (1.39)	5.11 (1.35)	5.11 (1.36)	5.10 (1.39)	5.13 (1.40)	0.394
Serum creatinine, mg/dl	0.72 (0.19)	0.72 (0.20)	0.73 (0.19)	0.73 (0.18)	0.73 (0.19)	0.70 (0.16)	0.441
eGFR, ml/min/1.73m^2^	78.6 (19.5)	78.2 (21.4)	77.2 (19.7)	78.6 (18.1)	79.5 (20.4)	81.5 (16.8)	0.236
HbA1c, %	7.26 (0.95)	7.22 (0.88)	7.26 (0.88)	7.27 (1.03)	7.34 (1.09)	7.15 (0.87)	0.413
Serum sodium, mEq/l	139.6 (1.8)	139.9 (1.9)	139.7 (1.9)	139.5 (1.8)	139.2 (1.9)	139.5 (1.6)	0.003
Serum potassium, mEq/l	4.44 (0.37)	4.31 (0.39)	4.42 (0.36)	4.48 (0.39)	4.50 (0.37)	4.51 (0.31)	<0.001
Serum chloride, mEq/l	104.3 (2.4)	104.4 (2.7)	104.6 (2.3)	104.2 (2.3)	104.0 (2.2)	104.1 (2.2)	0.03
Total cholesterol, mg/dl	198.8 (34.7)	197.1 (35.1)	201.8 (34.8)	198.3 (34.3)	198.2 (35.6)	196.6 (33.6)	0.425
HDL cholesterol, mg/dl	53.3 (14.0)	54.1 (14.5)	52.9 (13.6)	52.4 (13.4)	53.3 (13.1)	55.2 (15.9)	0.309
24h urinary albumin excretion, mg/day	44.4 (117.9)	41.5 (101.3)	57.7 (163.3)	34.7 (81.7)	34.7 (104.8)	50.6 (87.6)	0.080
24h urinary sodium excretion, g/day	4.50 (1.64)	3.51 (1.24)	4.23 (1.36)	4.63 (1.40)	4.99 (1.61)	5.87 (2.10)	<0.001

Categorical variables are expressed as the number (%) and continuous variables are expressed as the mean (SD).

BMI, body mass index; BP, blood pressure; CVD, cardiovascular disease; RAAS, renin-angiotensin-aldosterone system; eGFR, estimated glomerular filtration rate; HbA1c, glycated hemoglobin; HDL, high-density lipoprotein

### Sodium excretion

**Fig 2:**
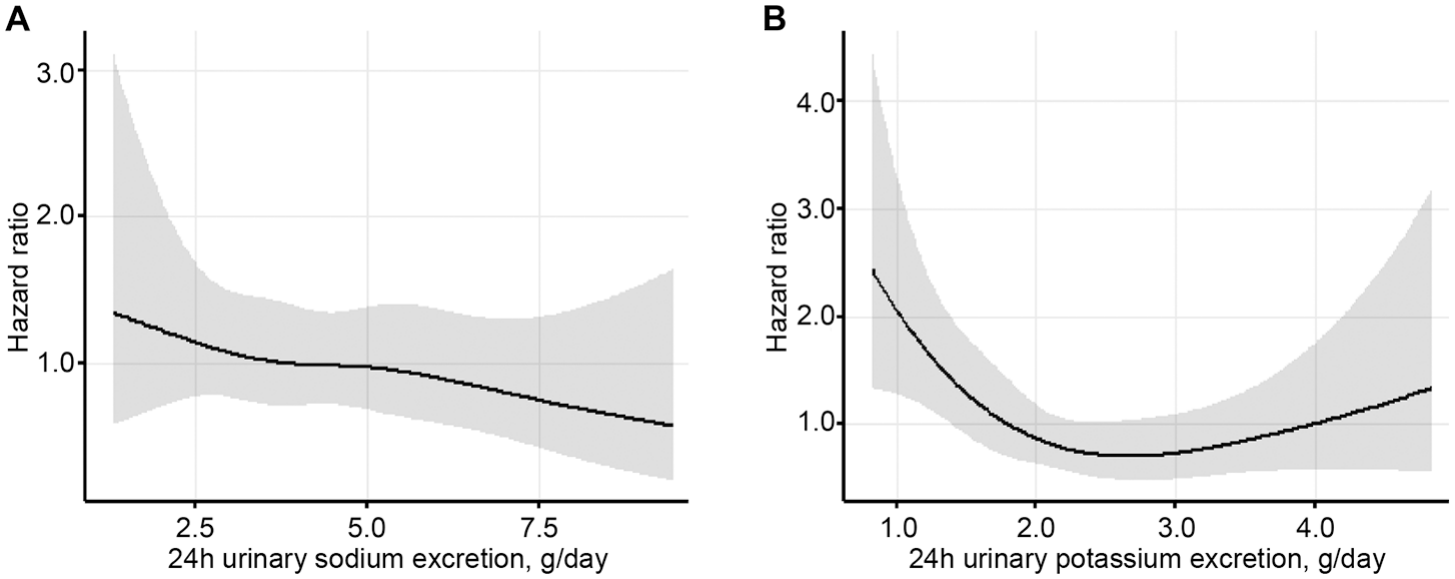
Association between 24h (A) urinary sodium and (B) potassium excretion and hazard ratio for 30% decline in eGFR or death. Multivariable spline regression analyses of hazard ratios and 95% confidence intervals of 30% decline of eGFR or death, adjusted for age, sex, body mass index, history of CVD, diabetic retinopathy, blood pressure, HbA1c, eGFR, uric acid, total cholesterol, high density lipoprotein, 24h urinary albumin excretion and (A) 24h urinary potassium excretion or (B) 24 urinary sodium excretion.

When compared with the reference category of <3.0 g/day, the univariate cox regression model showed that 3.0–4.0 g/day and 5.0–6.0 g/day were significantly associated with a reduced risk of 30% decline in eGFR or death (hazard ratio [HR], 0.55 and 0.51; 95% confidence interval [CI], 0.31 to 0.95 and 0.28 to 0.90; respectively). However, this difference became nonsignificant when a multivariate cox regression model was applied (Table 3 and Fig 2).

**Table 3:** Association between 24h urinary sodium excretion and 30% decline in eGFR and death.

	24h urinary sodium excretion				
Variable	<3.0 g/day	3.0–4.0 g/day	4.0–5.0 g/day	5.0–6.0 g/day	>6.0 g/day
Number of patients	220	274	333	222	181
Follow up duration, years	5.23 (2.31)	5.39 (2.35)	5.74 (2.21)	5.49 (2.31)	5.34 (2.23)
30% decline in eGFR or death, n (%)	30 (13.6)	22 (8.0)	46 (13.8)	17 (7.7)	15 (8.2)
Death, n	0	2	1	2	1
Analysis, HR (95% CI)					
Univariate analysis	1.00	0.55 (0.31–0.95)	0.84 (0.53–1.34)	0.51 (0.28–0.90)	0.59 (0.31–1.10)
Multivariate analysis	1.00	0.65 (0.37–1.14)	1.14 (0.69–1.88)	0.56 (0.30–1.06)	1.00 (0.49–2.04)
Multivariate analysis excluding A	1.00	0.63 (0.28–1.39)	1.02 (0.50–2.10)	0.35 (0.11–1.13)	1.18 (0.45–3.09)
Multivariate analysis excluding A or B	1.00	0.61 (0.22–1.65)	0.78 (0.31–1.98)	0.13 (0.01–1.15)	0.99 (0.30–3.31)
Multivariate analysis excluding C	1.00	0.38 (0.11–1.28)	1.28 (0.53–3.11)	0.58 (0.16–2.06)	0.84 (0.16–4.44)

Categorical variables are expressed as number (%) and continuous variables are expressed as mean (SD).

Multivariate analysis adjusted for age, sex, body mass index, history of CVD, diabetic retinopathy, blood pressure, HbA1c, eGFR, uric acid, total cholesterol, high density lipoprotein, 24h urinary albumin excretion and 24h urinary sodium excretion.

^A^: eGFR < 60 ml/min/1.73m^2^ or 24h urinary albumin excretion > 30 mg/day at baseline

^B^: A and history of CVD at baseline

^C^: eGFR > 60 ml/min/1.73m^2^

eGFR; estimated glomerular filtration rate; HR, hazard ratio, CI, confidence interval; CVD, cardiovascular disease; HbA1c, glycated hemoglobin

### Potassium excretion

When compared with the reference category of <1.5 g/day, 2.0–2.5 g/day and 2.5–3.0 g/day categories were significantly associated with a lower risk of 30% decline in eGFR or death in both univariate cox regression (hazard ratio [HR], 0.44 and 0.36; 95% confidence interval [CI], 0.26 to 0.74 and 0.19 to 0.70; respectively) and multivariate (HR, 0.49 and 0.44; 95% CI, 0.28 to 0.84 and 0.22 to 0.87; respectively) models. In contrast, categories 1.5–2.0 g/day and >3.0 g/day were nonsignificant in both univariate (HR, 0.71 and 0.70; 95% CI, 0.45 to 1.11 and 0.39 to 1.26; respectively) and multivariate (HR, 0.69 and 0.71; 95% CI, 0.43 to 1.10 and 0.37 to 1.34; respectively) models (Table 4 and Fig 2).

**Table 4:** Association between 24h urinary potassium excretion and 30% decline in eGFR and death.

	24h urinary potassium excretion				
Variable	<1.5 g/day	1.5–2.0 g/day	2.0–2.5 g/day	2.5–3.0 g/day	>3.0 g/day
Number of patients	242	349	301	194	144
Follow up duration, years	5.16 (2.31)	5.44 (2.32)	5.69 (2.29)	5.49 (2.29)	5.59 (2.08)
30% decline in eGFR or death, n (%)	36 (14.8)	41 (11.7)	24 (7.9)	12 (6.1)	17 (11.8)
Death, n	2	0	3	0	1
Analysis, HR (95% CI)					
Univariate analysis	1.00	0.71 (0.45–1.11)	0.44 (0.26–0.74)	0.36 (0.19–0.70)	0.70 (0.39–1.26)
Multivariate analysis	1.00	0.69 (0.43–1.10)	0.49 (0.28–0.84)	0.44 (0.22–0.87)	0.71 (0.37–1.34)
Multivariate analysis excluding A	1.00	0.63 (0.33–1.20)	0.33 (0.15–0.72)	0.27 (0.09–0.76)	0.62 (0.24–1.58)
Multivariate analysis excluding A or B	1.00	0.63 (0.27–1.49)	0.28 (0.10–0.77)	0.15 (0.03–0.74)	0.73 (0.22–2.40)
Multivariate analysis excluding C	1.00	0.49 (0.17–1.40)	1.03 (0.35–3.06)	0.86 (0.25–2.94)	1.66 (0.42–6.54)

Categorical variables are expressed as number (%) and continuous variables are expressed as mean (SD).

Multivariate analysis adjusted for age, sex, body mass index, history of CVD, diabetic retinopathy, blood pressure, HbA1c, eGFR, uric acid, total cholesterol, high density lipoprotein, 24h urinary albumin excretion and 24h urinary sodium excretion.

^A^: eGFR < 60 ml/min/1.73m^2^ or 24h urinary albumin excretion > 30 mg/day at baseline

^B^: A and history of CVD at baseline

^C^: eGFR > 60 ml/min/1.73m^2^

eGFR; estimated glomerular filtration rate; HR, hazard ratio, CI, confidence interval; CVD, cardiovascular disease; HbA1c, glycated hemoglobin

### Subgroup analysis

In subgroup analysis limited to patients without CKD or those without CKD and CVD, the same associations between 24h urinary sodium and potassium excretion and outcomes were observed. In patients with an eGFR < 60 ml/min/1.73m^2^, no significant associations were found (Tables 3 and 4). When the sodium to potassium ratio was expressed as a continuous variable, a cox regression model showed that association with the outcome was nonsignificant in both univariate (HR, 1.15; 95% CI, 0.92 to 1.36) and multivariate (HR, 1.15; 95% CI, 0.94 to 1.41) models.

## Discussion

This retrospective study showed that 24h urinary sodium excretion was not significantly associated with either a risk of a 30% decline in eGFR or death in patients with diabetes. However, 24h urinary potassium excretion < 1.5 g/day was found to be significantly associated with an increased risk of a 30% decline in eGFR or death, when compared with the categories of 2.0–2.5 g/day and 2.5–3.0 g/day. Although this relationship was not significant, it does suggest a higher risk when compared with 24h urinary potassium excretion of 1.5–2.0 g/day and >3.0 g/day. This association was found to be independent of additional factors that could affect kidney outcomes, including albuminuria.

With reference to urinary sodium excretion, no significant associations with outcomes were observed in this study. The results of previous studies that have examined the association between urinary sodium excretion and kidney outcomes were largely nonsignificant by multivariate analysis, even where these studies were heterogeneous with respect to study design, participants’ backgrounds, or outcomes definitions. Among them, He et al.[17] reported an association between increased 24h urinary sodium excretion and a high risk for kidney outcomes in their main analysis. However, these associations were found to be nonsignificant after adjustment for proteinuria. That study noted that BP and proteinuria were intermediate variables on the causal pathway for CKD progression, and therefore, did not adjust for them during primary analysis. The present study included albuminuria and BP in multivariate analysis, as dietary salt loading in an animal model has been reported to cause deterioration of GFR and increased proteinuria, independent of BP[22]. Sodium restriction has been found to reduce albuminuria, even when a clinically significant reduction in BP in patients with CKD has not been achieved[23]. Indeed, numerous previous studies have conducted multivariate analysis adjusted for albuminuria or proteinuria [8,10–12,16,17,24]. The results of the present study were consistent with these reports; however, a significant association may have been overlooked, due to the small number of patients analyzed.

As for potassium excretion, the results of this study largely correspond with past reports. A post hoc analysis of randomized clinical trials included 28 879 patients and indicated that a higher 24h urinary potassium excretion, as estimated by a fasting morning urine sample, was associated with lower odds of kidney outcomes (defined as a 30% decline in eGFR or chronic dialysis)[8]. Another report from a single-center, observational cohort study included 623 patients with type 2 diabetes and normal kidney function and found that higher urinary potassium excretion was associated with a slower decline in renal function and lower incidence of composite outcomes of kidney and CVD events[16]. In contrast, the Chronic Renal Insufficiency Cohort (CRIC) study showed that higher 24h urinary potassium excretion was associated with an increased risk for CKD progression, which was defined as a halved eGFR and ESRD. However, this association diminished after adjusting for proteinuria and no significant association was observed according to the amount of potassium excreted[17]. The results of our study correspond with two of the aforementioned studies; however, they are not in line with the results obtained in the CRIC study. This could be attributable to the mean eGFR of patients analyzed in both the present study and the corresponding studies by Smyth et al.[8] and Araki et al.[16], as this value was not as low as that of the CRIC study (mean eGFR: 43 ml/min/1.73m^2^). Indeed, in the present study, a subgroup analysis of patients with an eGFR < 60 ml/min/1.73m^2^ found no significant association between potassium excretion and outcomes. However, at this stage, conclusions should be withheld, considering the small number of patients concerned. Further study is needed in order to clarify the association between high urinary potassium excretion and kidney outcomes in patients with CKD.

The present study showed that potassium was significantly associated with kidney outcomes, whereas the sodium to potassium ratio was not. The trial of hypertension prevention follow up study showed that a higher sodium to potassium was associated with an increased risk of subsequent CVD in patients with prehypertension[25]. In contrast, recent reports in patients with type 2 diabetes and normal kidney function have shown that a high sodium to potassium was associated with a high risk of composite outcomes of kidney and CVD events[16]. However, that study did not assess the independent association with risk of kidney outcomes. The results of the present study suggest that sodium to potassium ratio may not be associated with kidney outcomes.

The inconsistent results reported in previous studies are because of several potential reasons. First, patients with a reduced eGFR may possibly present with altered potassium handling by the kidneys (reduced potassium filtration or tubular secretion)[26] or increased extrarenal excretion (such as, by the gastrointestinal route)[27]. Thus, urinary excretion may not reflect dietary potassium intake in patients with CKD. Secondly, high urinary potassium excretion reflects a high dietary potassium intake and can lead to hyperkalemia, which could result in increased risk of mortality or major cardiovascular events[28]. Finally, patients with CKD are often prescribed renin-angiotensin-aldosterone blockers, which could affect potassium homeostasis[29]. While all of these factors could confound results, high urinary potassium excretion in patients with diabetes is likely associated with reduced risk for kidney outcomes.

Several protective effects of potassium on kidney function have been reported. In animal models, increased serum potassium levels cause decreased vascular resistance[30], while potassium supplementation can reduce renal inflammation[31]. In humans, increased potassium intake reduces BP[32] and has a beneficial effect on the risk of CVD[8,33]. In addition, a high-potassium diet is generally equivalent to a healthy diet[34] containing many fruits and vegetables, which has been reported to improve kidney outcomes[12].

Compared with previous reports, this study design had several valuable strengths [8,10,12,16,17]. Firstly, a 24h urinary collection test was conducted in all patients with diabetes. Second, the accuracy of urine collection for analysis was ensured by urinary creatinine excretion, as opposed to self-reporting by patients. Notably, 1078 of 3793 patients with an eGFR > 30 ml/min/1.73m^2^ were excluded from this study, as their 24h urinary creatinine deviated by ±25% of the predicted value (Fig 1). Finally, kidney outcomes were not based not on the progression of albuminuria or proteinuria, but rather on a 30% decline in eGFR.

In addition, this study had several limitations that warrant discussion. First, this was a retrospective study design and a selection bias did exist, in that only patients who had received a 24h urine collection test were included. Second, we may have overestimated or, indeed, overlooked the associations with outcomes, as an insufficient number of patients were included in the analysis. Third, treatment content or BP and glycemic control during the follow up period were not considered. Finally, a single 24h urine collection test is insufficient for estimating a patient’s usual dietary status[35].

In conclusion, this retrospective study showed that 24h urinary sodium excretion was not significantly associated with either a risk of 30% decline in eGFR or death in patients with diabetes; whereas, 24h urinary potassium excretion less than 1.5 g/day was significantly associated with increased risk compared with 2.0–2.5 g/day and 2.5–3.0 g/day. Larger, prospective studies will elucidate the association between higher sodium or potassium intake and an increased or decreased risk of kidney outcomes in patients with diabetes.

## Supporting Information

S1 Table Anonymous data set of 1230 patients with diabetes. (XLSX)
